# A Subdural Hygroma Necessitating a Subdural-Peritoneal Shunt in a Pediatric Patient Following Total Cranial Vault Remodeling Surgery

**DOI:** 10.7759/cureus.47348

**Published:** 2023-10-19

**Authors:** Lindsey Hunt, Rasha G Elbadry, Andrea Ray, Tanya Minasian

**Affiliations:** 1 Neurosurgery, Loma Linda University School of Medicine, Loma Linda, USA; 2 Neurosurgery, Loma Linda University Medical Center, Loma Linda, USA; 3 Plastic and Reconstructive Surgery, Loma Linda University Medical Center, Loma Linda, USA; 4 Neurosurgery, Loma Linda University Health, Loma Linda, USA

**Keywords:** pediatric neurosurgery, complication of treatment, shunt placement, subdural hematoma (sdh), sagittal craniosynostosis

## Abstract

Sagittal synostosis is a common non-syndromic synostosis treated with open or endoscopic cranial vault remodeling. Early intervention is recommended to avoid restricted brain growth, increased intracranial pressure, and resultant developmental delay. Common complications such as failure or reconstruction, cerebrospinal fluid leak, blood loss, and stroke are well-reported in the literature. Here, we present a rare case of the development of a subdural hygroma following cranial vault remodeling in a seven-month-old male, necessitating the insertion of a subdural-peritoneal shunt.

## Introduction

Sagittal synostosis is the most common non-syndromic synostosis affecting pediatric patients, with an incidence of 45% [1]. A concern for early suture fusion includes impeded brain growth and the potential of developing increased intracranial pressure (ICP) [2]. Baseline papilledema in patients with scaphocephaly has been reported in 10% of cases. Postoperative papilledema in single-suture sagittal craniosynostosis after cranial vault remodeling has been reported in 5% of cases [3]. Renier et al. described elevated ICP in 14% of patients with scaphocephaly preoperatively, with much higher percentages reported in multi-suture craniosynostosis (38% brachycephaly; 60% oxycephaly; 47% complex craniosynostosis), with up to 35% of syndromic patients having elevated ICP post-cranial vault remodeling [4].

There is some heterogeneity in how sagittal craniosynostosis is managed in non-syndromic patients, primarily based on surgeon preference and patient factors [5]. The Craniosynostosis: Developing Parameters for Diagnosis, Treatment, and Management Conference 2011 recommended treating sagittal synostosis before one year of age [6]. Treatment options include the pi procedure, subtotal cranial vault remodeling with calvarial reconstruction, whether open or endoscopic, total cranial vault remodeling, and options based on presentation and surgeon preference.

Known complications from cranial vault remodeling surgery include failure of reconstruction (re-ossification, contour irregularities, dissatisfaction with cosmetic result), bleeding, cerebrospinal fluid (CSF) leak, meningitis, stroke, and mortality [7]. A subdural hygroma is a rare complication from barrel stave osteotomy surgery. Theories for the causes of chronic subdural hematomas range from intracranial hypotension, arachnoid cyst development, anomalies in venous anatomy, slow bleeding from cortical veins, cortical ischemia, and idiopathic causes.

Here, we present a case of non-syndromic sagittal synostosis following uncomplicated total cranial vault remodeling, with immediate postoperative development of increased ICP, ultimately necessitating a subdural-peritoneal shunt for long-term management.

## Case presentation

**Figure 1:**
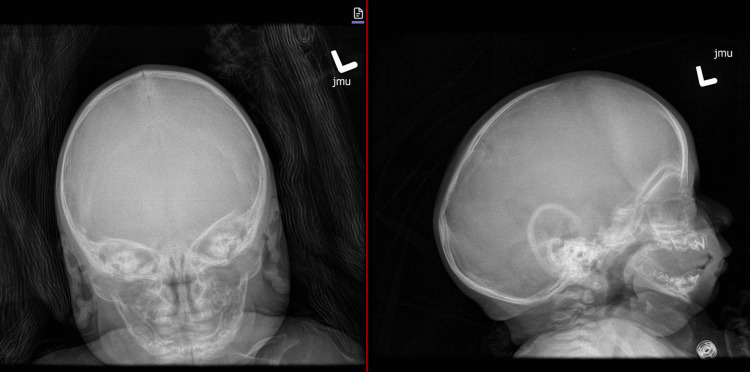
Skull X-ray obtained at the age of 15 weeks showing sagittal synostosis.

**Figure 2:**
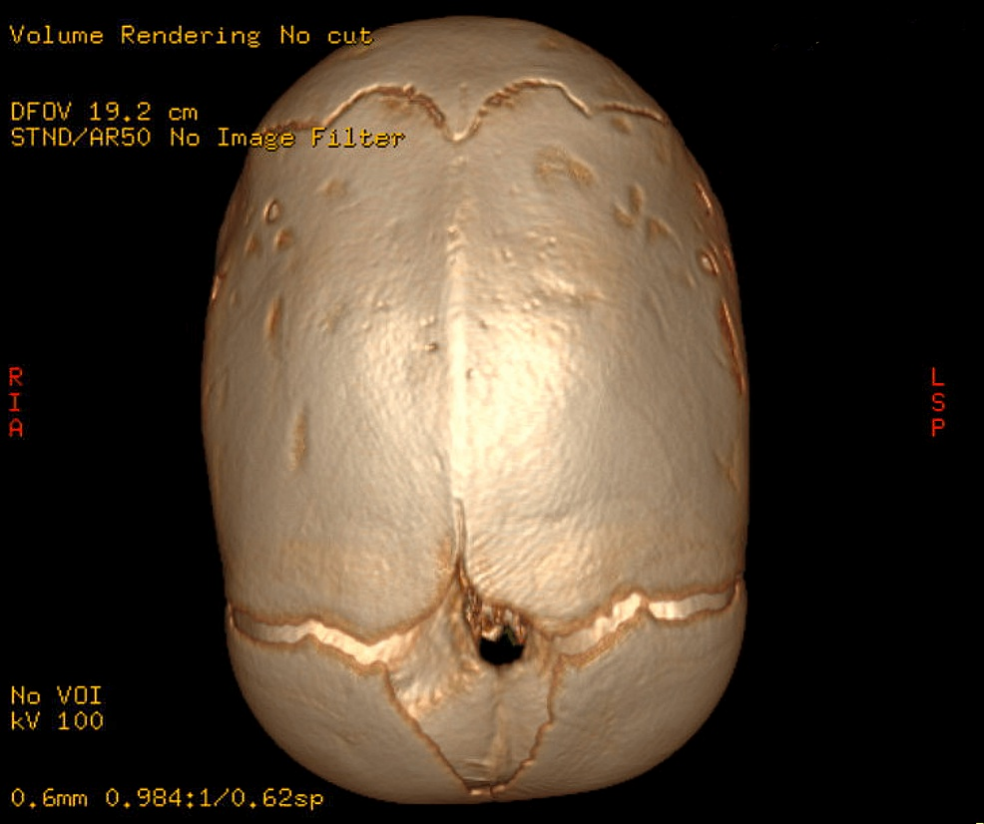
CT of the head showing sagittal synostosis.

An eight-week-old male presented to the emergency department with a history of cough, congestion, and rhinorrhea for two days. On admission, his head size was noted to be in the 94th percentile for his age. A skull X-ray was obtained and demonstrated sagittal synostosis. He was referred to our craniofacial clinic and was initially assessed when he was 15 weeks old (Figure 1). The parents of the child noted that he sometimes had some horizontal nystagmus that intermittently appeared and resolved but denied any increased irritability, neurologic deficits, or seizure-like activity. Baseline ophthalmology evaluation demonstrated astigmatism without evidence of papilledema at four months of age. A CT of the head was obtained and confirmed the diagnosis of sagittal synostosis (Figure 2). At seven months of age, he underwent a sagittal strip craniectomy, barrel stave osteotomies, and bilateral frontal wedge osteotomies for total cranial vault remodeling. There were no dural violations and no venous sinus injury. Procedures to prevent blood loss intraoperatively included incision infiltration with 0.25% marcaine plus 1:100,000 epinephrine, careful dissection, and the use of Ostene (a bone hemostasis agent) to stop bleeding from the cranium. The patient was transfused intraoperatively and did not need transfusion postoperatively. The patient recovered well and had no immediate postoperative complications.

**Figure 3:**
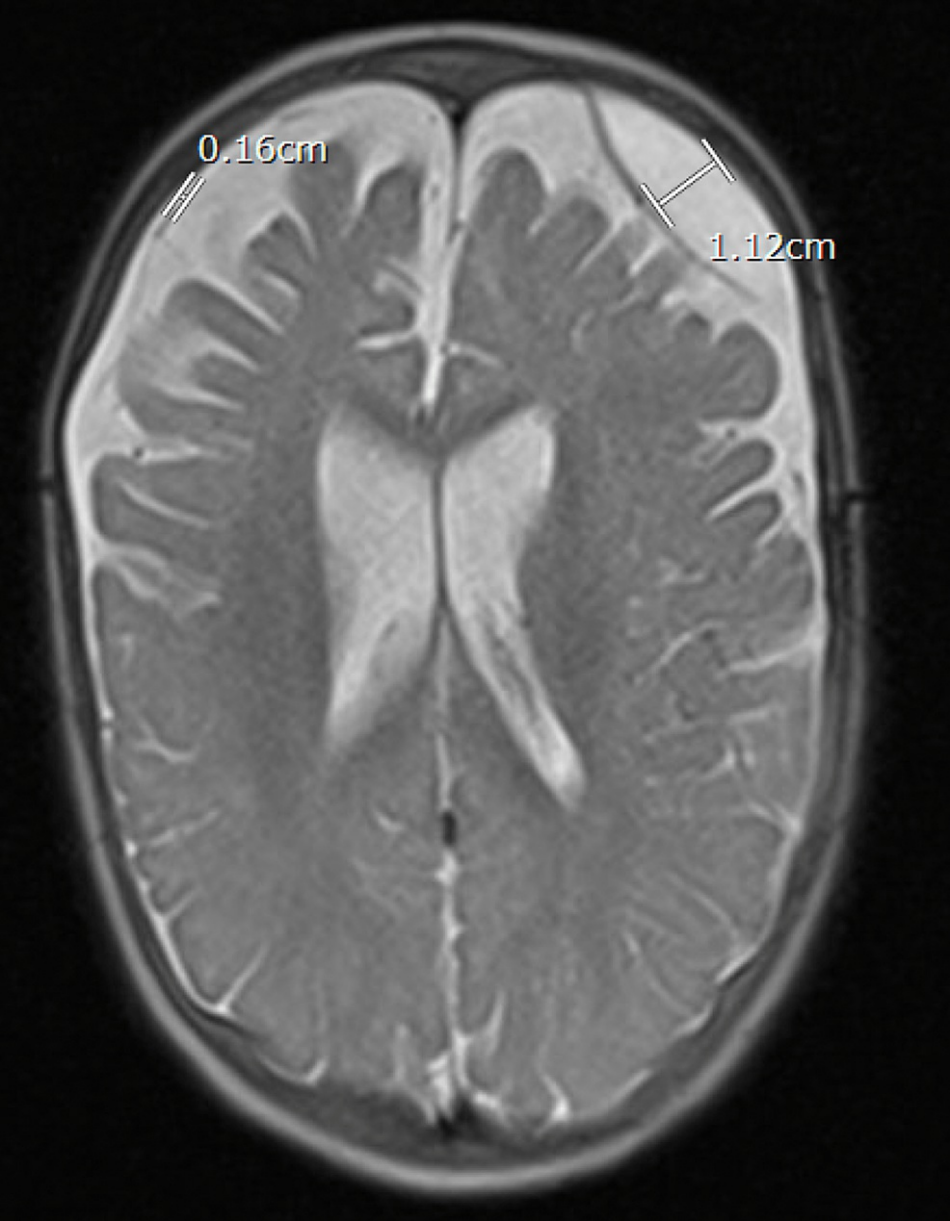
MRI of the brain T2 axial view showing bilateral subdural hygroma.

One month later, at eight months old, the patient was re-evaluated by ophthalmology and found to have bilateral papilledema, a new finding. A limited MRI of the brain was obtained and demonstrated bilateral subdural fluid collections, left greater than right (Figure 3). The parents noted that the patient had been more active recently, received a few falls onto his head on the tile while crawling, and was more irritable, but no vomiting, seizures, or other gross neurologic abnormality was noted. On examination, he was grossly intact with reactive equal pupils bilaterally. The decision was made to evacuate the bilateral extra-axial fluid collections via bilateral burr holes. Bilateral subdural hygromas were encountered under high pressure, with notable re-expansion of the brain cortex after decompression. A drain was inserted in the subdural space on the left.

**Figure 4:**
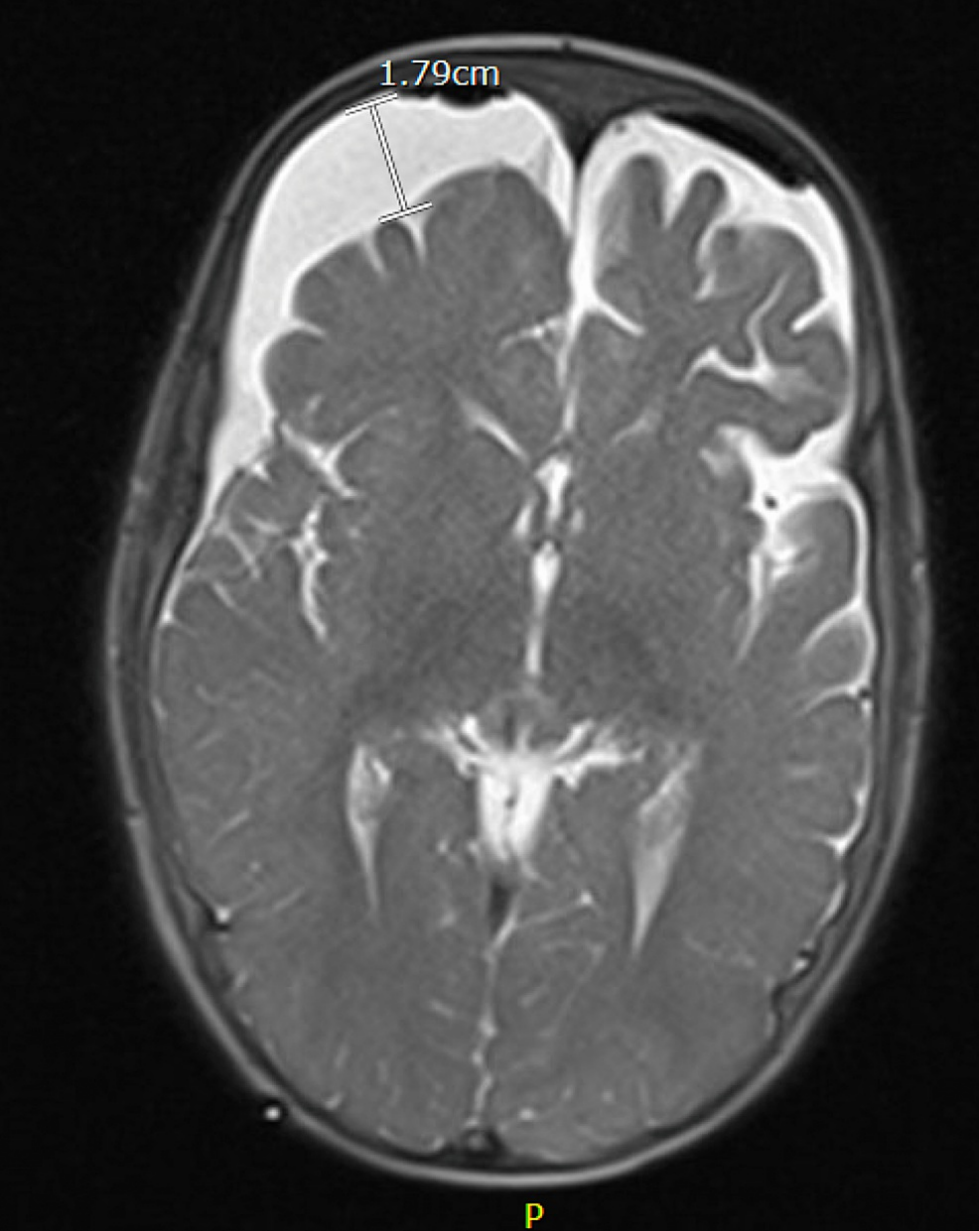
MRI of the brain T2 axial view showing persistent subdural collection, right greater than left, status post-clamping trial.

**Figure 5:**
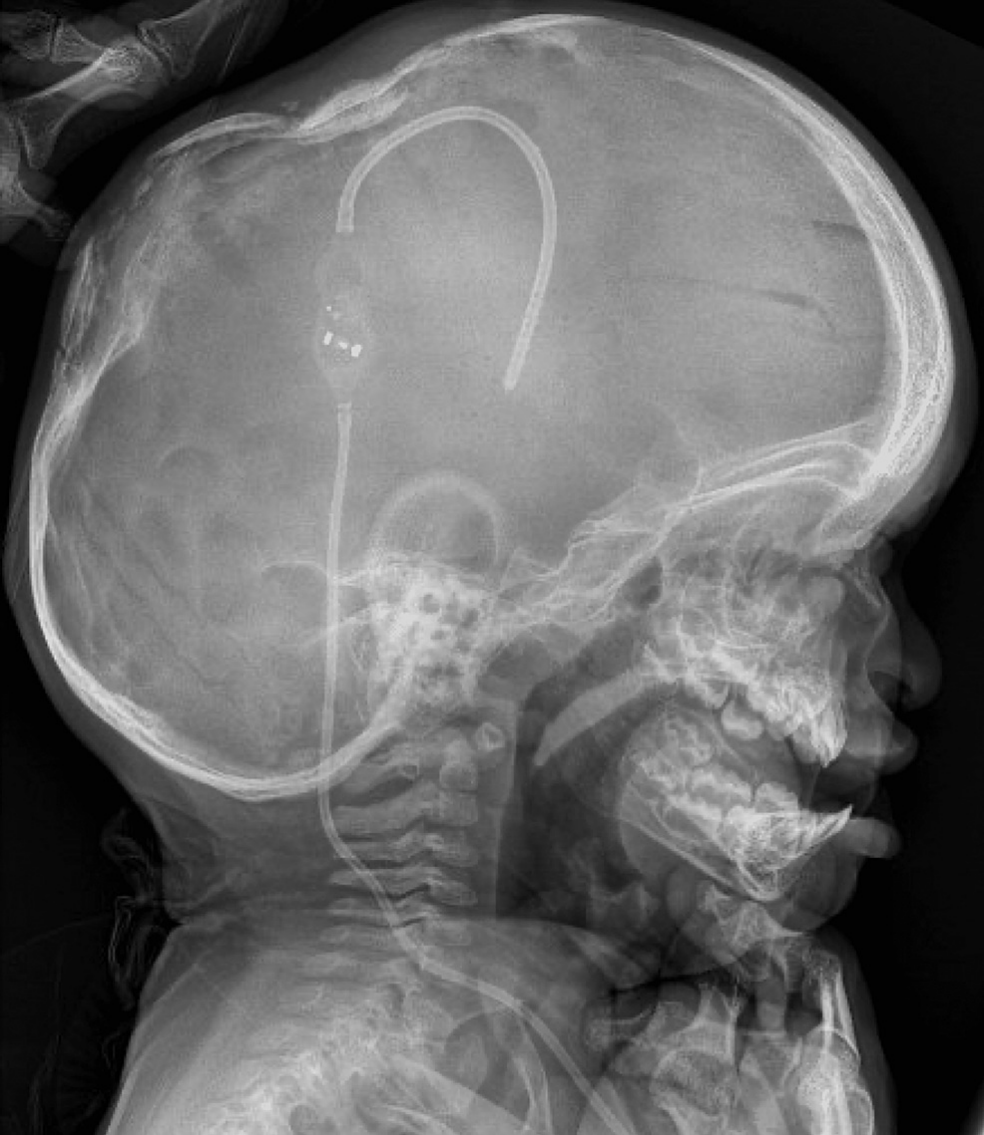
Skull X-ray showing the insertion of a programmable subdural-peritoneal shunt.

**Figure 6:**
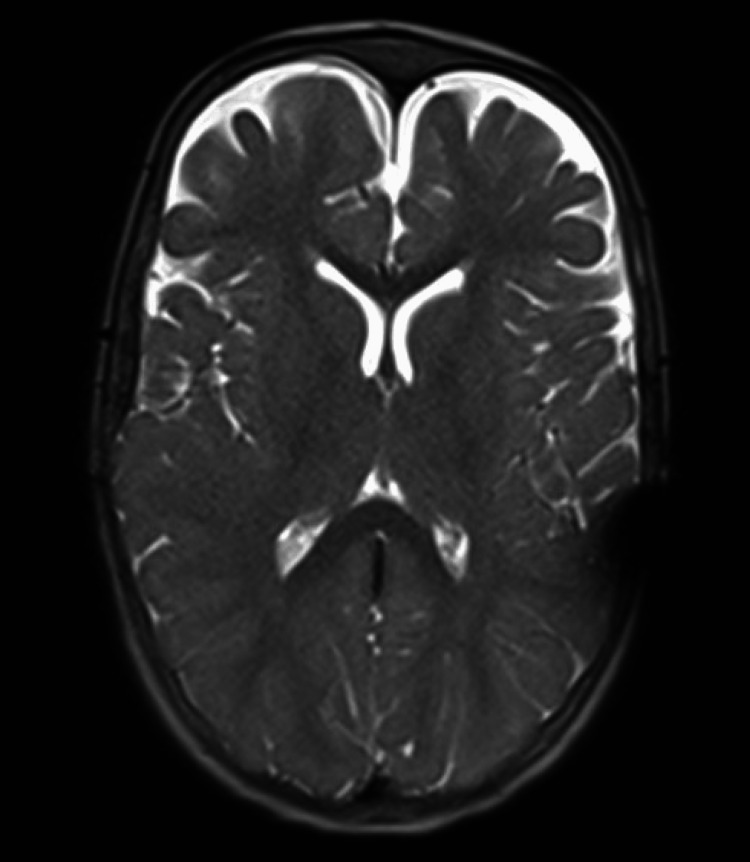
Postoperative MRI showing interval improvement of bilateral subdural hygroma after the insertion of a subdural-peritoneal shunt.

In subsequent days, two subdural drain clamp trails were attempted, both unsuccessful. This was anticipated given the very high drain outputs. When the drain was clamped, he became irritable, had a tense fontanelle, and developed tense subgaleal fluid collections bilaterally (Figure 4). Given the failed weaning trials, the decision was made to place a unilateral subdural-peritoneal shunt with a programmable valve (Figure 5). Three months postoperatively, ophthalmology re-evaluated him and noted the resolution of papilledema. Imaging postoperatively demonstrated resolution of subdural hygromas bilaterally (Figure 6). The patient is meeting all milestones and is neurologically intact.

## Discussion

The presentation of chronic subdural hygroma following cranial vault surgery is a rare complication among pediatric patients. Only a few case studies have documented this complication. In one case report, a four-year-old male with severe dolichocephalic deformity underwent lateral and posterior cranial vault remodeling for sagittal synostosis. A routine follow-up CT scan demonstrated a left-sided chronic subdural hematoma with a 1.5 cm midline shift and subfalcine herniation. The patient underwent left-sided surgical evacuation and placement of a subdural drain. The authors noticed that the subdural hematoma was under high pressure. They theorized that the unique venous anatomy of significant egression through large emissary veins may have contributed to this patient’s presentation [8]. No papilledema was noted on this patient’s clinical examination before either intervention.

In another case report, a four-month-old male infant was diagnosed with premature fusion of bilateral coronal and anterior sagittal sutures. The cranial vault remodeling surgery involved a bifrontal craniotomy, frontal-orbital advancement, and barrel stave osteotomies. Remodeling was complicated by a small dural tear repaired with a small dural patch. Three months following surgery, the patient was noted to have an abnormal head contour, diffuse swelling was palpated between craniotomy edges, the neurologic examination was grossly normal, and there was no papilledema. CT scan revealed a hypodense fluid collection in the bilateral frontal area, no midline shift, and no mass effect. The fluid collection was tapped, and clear fluid egressed from the cavity. The patient was followed with serial ultrasound studies, gradual resolution of the fluid collections occurred, and no further intervention was required [9]. In a retrospective case review of 44 patients with sagittal synostosis, only one patient had a subgaleal hematoma requiring postoperative drainage. This retrospective review also highlighted the improved morbidity for cranial vault remodeling compared to prior studies, lower complication rates, decreased blood loss, and shorter operative times [10].

Our case is also unique because of the pediatric presentation of subdural hygroma following surgery. In a one-year, cross-sectional study, trauma was the most common cause of subdural hematoma and effusion for children [11]. In a 19-patient retrospective review, non-traumatic spontaneous subdural hematomas in pediatric patients were associated with comorbidities such as acute myeloid leukemia, a history of shunt operations, epilepsy, mucopolysaccharidosis, subdural effusion, autism, coagulopathies, cardiac defects, Marfan’s syndrome, and late neonatal sepsis [12].

A retrospective, single-center review found previous shunts to be the most common predisposing factor to subdural hygroma, followed by a history of trauma. Interestingly, recurrent cases of chronic subdural hematoma were excluded from the study, and no cases of chronic subdural hematoma following craniosynostosis remodeling surgery were recorded [13]. Subdural hygromas are a known complication of endoscopic third ventriculostomies and fenestration of arachnoid cysts for patients less than one year of age. The mechanism is theorized to be a sudden decrease in ventricular volume secondary to CSF drainage during endoscopic third ventriculostomy [14]. Particularly in pediatric patients, non-accidental trauma can cause subdural hematoma to progress to chronic subdural hygroma. In a population-based case series, subdural hemorrhages were found to be associated with non-accidental trauma and required a complete and thorough workup [15].

The mechanism for why subdural hygromas form has been hypothesized based on multiple studies from both adult and pediatric populations. In adults, subdural hygroma formation is associated with advanced age, male sex, and aneurysm location with arachnoid dissection following aneurysm surgery [16]. In adult patients, subdural hygromas can form after endoscopic third ventriculostomy procedures [17]. Spontaneous intracranial hypotension diagnosed based on history, physical examination, and CSF pressure on lumbar puncture has been associated with spontaneous subdural hematomas in adults. The mechanism behind the development of chronic subdural hematoma is theorized to be secondary to changes in CSF fluid pressure, cushion effects, and dynamics [18]. A retrospective cohort study classified traumatic subdural hematoma based on CSF dynamics. Specifically, there was no impairment of CSF absorption, external hydrocephalus with mild-to-moderate CSF absorption impairment, and marked mass effect secondary to impaired reabsorption [19].

Arachnoid cysts can precede or complicate the clinical course for younger patients with chronic subdural hematomas [20]. The main mechanism of fluid accumulation is postulated to be via effusion [21]. Only 0.6% developed peri-cerebral fluid collections requiring subdural peritoneal shunt. This can be explained partially because of the increased volume of the cranial vault but no corresponding change in brain volume, likely why the cavity fills with CSF.

## Conclusions

Subdural hygromas are a possible, albeit rare, complication of total cranial vault remodeling surgery. This case report is unique for subdural hygroma formation without any obvious predisposing factors. Moreover, the occurrence of papilledema immediately postoperatively with the necessitation of a subdural-peritoneal shunt highlights the urgency and importance of treating this complication. This case also demonstrates the critical importance of institutional craniofacial clinics, where patients are followed closely in the perioperative period, with specialists monitoring the patients for potential postoperative concerns. Further investigation into specific mechanisms behind subdural hygroma accumulation and resolution is warranted to improve the outcome and morbidity of these vulnerable patients.
